# Comparison of CT-guided suture hook anchor and indocyanine green localization for multiple pulmonary nodules

**DOI:** 10.3389/fonc.2026.1905816

**Published:** 2026-07-22

**Authors:** Yu-Tao Xian, Kai Wan, Tian-Tian An, Hai-Bo Dong

**Affiliations:** 1 Department of Radiology, Ningbo Medical Center Lihuili Hospital, Ningbo, China; 2 Department of Radiology, Xuzhou Central Hospital, Xuzhou, China

**Keywords:** CT, indocyanine Green, localization, pulmonary nodule, suture hook anchor

## Abstract

**Background:**

The preoperative application of computed tomography (CT), particularly suture hook anchor (SHA) and indocyanine green (IG) injection, for guiding the localization of pulmonary nodules (PNs) is increasingly used. Nevertheless, these two approaches have not been compared in terms of safety and performance. Here, the effectiveness and safety of CT-guided SHA and IG localization for multiple PNs during video-assisted thoracoscopic surgery (VATS) were evaluated.

**Methods:**

Consecutive cases with multiple PNs that received SHA or IG under CT guidance before VATS between January 2022 and December 2024 were retrospectively analyzed. Localization success, procedure duration, complication rates, and VATS-related outcomes analyzed.

**Results:**

Seventy-four cases were enrolled, of which 36 (79 nodules) underwent SHA localization, and 38 (92 nodules) underwent IG localization. All patients successfully underwent one-stage localization and subsequent VATS resection. The technical success rates for both procedures were 100%. The IG-mediated procedure was markedly shorter than SHA (10.6 ± 4.4 min vs. 23.4 ± 11.9 min, P = 0.001). The incidence of pneumothorax (27.8% vs. 36.8%, P = 0.405) and pulmonary hemorrhage (38.9% vs. 21.1%, P = 0.093) was comparable between the two procedures. VATS limited resections were technically successful in all patients (100% in both groups), with all nodules resected in a single operative session. The median operative duration was comparable between the SHA and IG groups (85 minutes vs. 80 minutes, P = 0.269).

**Conclusions:**

Both SHA and IG-mediated localization under CT guidance safe and effective for the preoperative evaluation of multiple PNs. While outcomes were similar across both methods, IG localization demonstrated the advantage of significantly reduced localization time, suggesting it may be a more time-efficient option in clinical practice.

## Introduction

Video-assisted thoracoscopic surgery (VATS) has become a widely adopted approach for treating high-risk pulmonary nodules (PNs). To enhance the accuracy and efficiency of VATS, preoperative localization under computed tomography (CT) guidance is frequently employed (1–3). These methods assist surgeons in precisely identifying nodule locations during resection, particularly when performing limited resections such as wedge or segmentectomy, thereby preserving healthy lung parenchyma (4).

Approximately 21% of patients with high-risk PNs present with multiple lesions (5). In such cases, surgical management often necessitates a broader resection field compared to the treatment of a single nodule. Consequently, accurate preoperative localization of each lesion is critical to minimize the extent of lung tissue resected and to optimize surgical outcomes.

Among available localization techniques, the suture hook anchor (SHA) has recently emerged as a viable alternative to the conventional hook-wire system (6–8). Unlike metallic hook-wires, SHA devices utilize a flexible tri-colored suture as the external marker, offering better patient comfort and potentially reducing the risk of complications (6–8). In addition to solid fixation devices like SHA, liquid-based localization methods, most notably, those using indocyanine green (IG), have also gained attention due to their procedural simplicity and low cost.

Despite the increasing use of both SHA and IG techniques, comparative studies assessing their safety and efficacy, particularly for localizing multiple PNs, remain limited. Therefore, the objective of this study was to retrospectively evaluate and compare the clinical effectiveness and safety profiles of CT-guided SHA and IG localization methods in patients undergoing one-stage VATS resection for multiple pulmonary nodules.

## Methods

### Study design

This retrospective cohort study was approved by the Ethics Committees of both Ningbo Medical Center Lihuili Hospital (No. 2023-019RS) and Xuzhou Central Hospital (No. XZXY-LJ-20241220-048). Written informed consent was not required as the investigation was retrospective.

Cases of multiple PNs treated with VATS following SHA or IG-mediated localization under CT guidance between January 2022 and December 2024 were enrolled. Eligibility criteria were as follows: (a) presence of two or more high-risk PNs; (b) all nodules located unilaterally; (c) maximum diameter ≤ 15 mm for solid nodules and ≤ 30 mm for ground-glass nodules. Exclusion criteria included: (a) distance between nodule and pleura > 4 cm; (b) nodule diameter < 6 mm; and (c) patient age younger than 18 or older than 80 years.

### Preoperative PN localization under CT guidance

In both groups, all target PNs were localized in a single CT-guided session. Procedures were conducted under local anesthesia. The choice of patient positioning and needle entry site was based on the anatomical position of the PNs. However, for patients with multiple PNs, different PNs had the different anatomical positions, therefore, patient’s body position may change between the different PN localization procedures within the same session because localization for different PNs may require different body positions.

In SHA, a SHA device (Senscure, Ningbo, China) was loaded into a 20-gauge needle for guidance. The needle was advanced through the chest wall into the lung parenchyma toward the target nodule. During insertion, multiple CT scans were performed to adjust the trajectory of the needle. When the tip of the needle was within 10 mm of the lesion, the release buckle was disengaged and the push rod was pressed to deploy the four-hook anchor. The needle was then withdrawn while the rod was advanced further to leave the tri-colored suture along the needle tract. A final CT scan confirmed the placement and checked for procedure-related complications.

For IG localization, a 21-gauge needle was used to access the lesion via a similar puncture approach. After the needle reached the desired position, approximately 0.3 ml of IG solution (2.5 mg/ml) was slowly injected, followed by smooth needle withdrawal. A post-procedural CT scan was then performed to assess for complications.

In both groups, a CT scan was conducted after the initial localization to exam the situation of the complications. If no pneumothorax occurred, the subsequent localization could be directly performed. If the pneumothorax was less than 2 cm, the puncture needle was placed into the pneumothorax area and a injector was connected to the puncture needle to extract the air in the chest cavity, the subsequent localization could be performed after the pneumothorax was resolved. If the pneumothorax was larger than 2 cm, a chest tube should be placed and a injector was connected to the chest tube to extract the air in the chest cavity, the subsequent localization could be performed after the pneumothorax was resolved.

### VATS resection

VATS resection was scheduled within 3 hours of localization. This involved an incision between 3 and 5 cm in the wall of the chest to accommodate the thoracoscopic instruments. In the SHA group, localization was guided by visualizing the tri-colored suture, while in the IG group, IG fluorescence on the pleural surface was used to guide resection. Wedge resection was performed for nodules located ≤ 2 cm from the pleural surface; otherwise, segmentectomy was chosen. All nodules were resected in a single operative session. Resected specimens were subjected to intraoperative pathological evaluation. If a lesion was found to represent invasive lung cancer, further lobectomy and dissection of the mediastinal lymph nodes were conducted. In cases with multiple invasive tumors, lobectomy was performed for lesions with the highest pathological stages.

### Outcome measures

For SHA, technical success represented (a) clear intraoperative visualization of the tri-colored suture and (b) absence of anchor dislodgement. For IG localization, success was defined as (a) visible IG fluorescence on the pleural surface during VATS and (b) absence of significant dye diffusion. Body position change was defined as the major repositioning (e.g., from supine to prone) on the CT table. The minor adjustments of the patient’s orientation was not considered as body position change. Successful limited resection represented removal of the entire nodule within the resected tissue.

The primary endpoint was defined as the technical success of the localization. The duration of localization, complication rates associated with localization, intraoperative VATS outcomes, and final pathological diagnoses represented secondary endpoints.

### Statistical analyses

Continuous variables are presented as mean ± standard deviation or as median with interquartile range (Q1:Q3), according to the data distribution. Intergroup comparisons were made with independent-sample t-tests or Mann–Whitney U tests. Categorical variables were analyzed with chi-square or Fisher’s exact tests. Risk factors for localization-related complications were evaluated with logistic regression analysis. A P-value < 0.05 was considered statistically significant. All data were analyzed using SPSS 16.0.

## Results

### Patients

**Figure 1:**
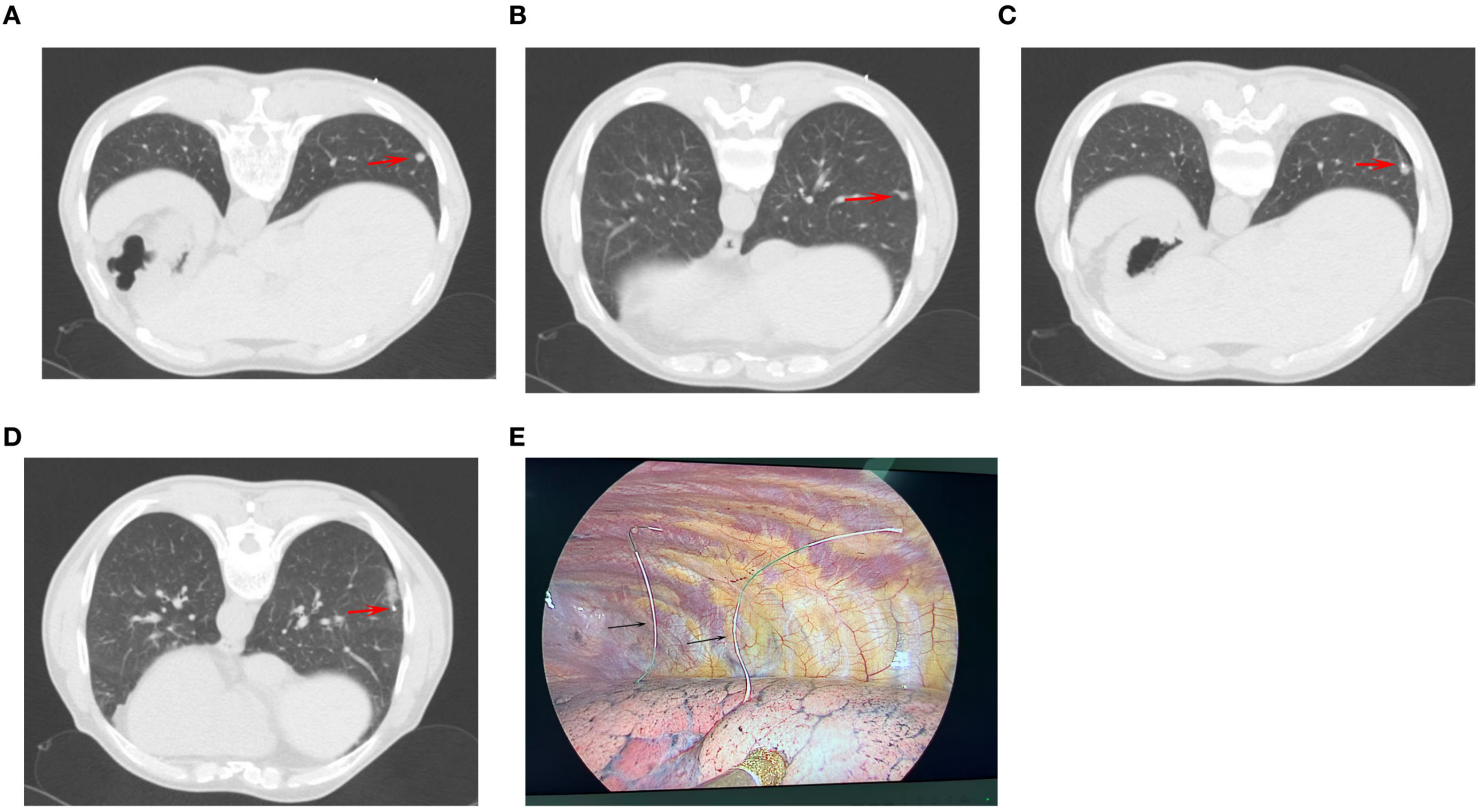
**(A, B)** The CT-guided SHA localization for PNs (arrows) in the right lower lobe. **(C, D)** The SHAs (arrows) were placed near the PNs for localization. **(E)** The SHAs (arrows) were detected during the VATS.

**Figure 2:**
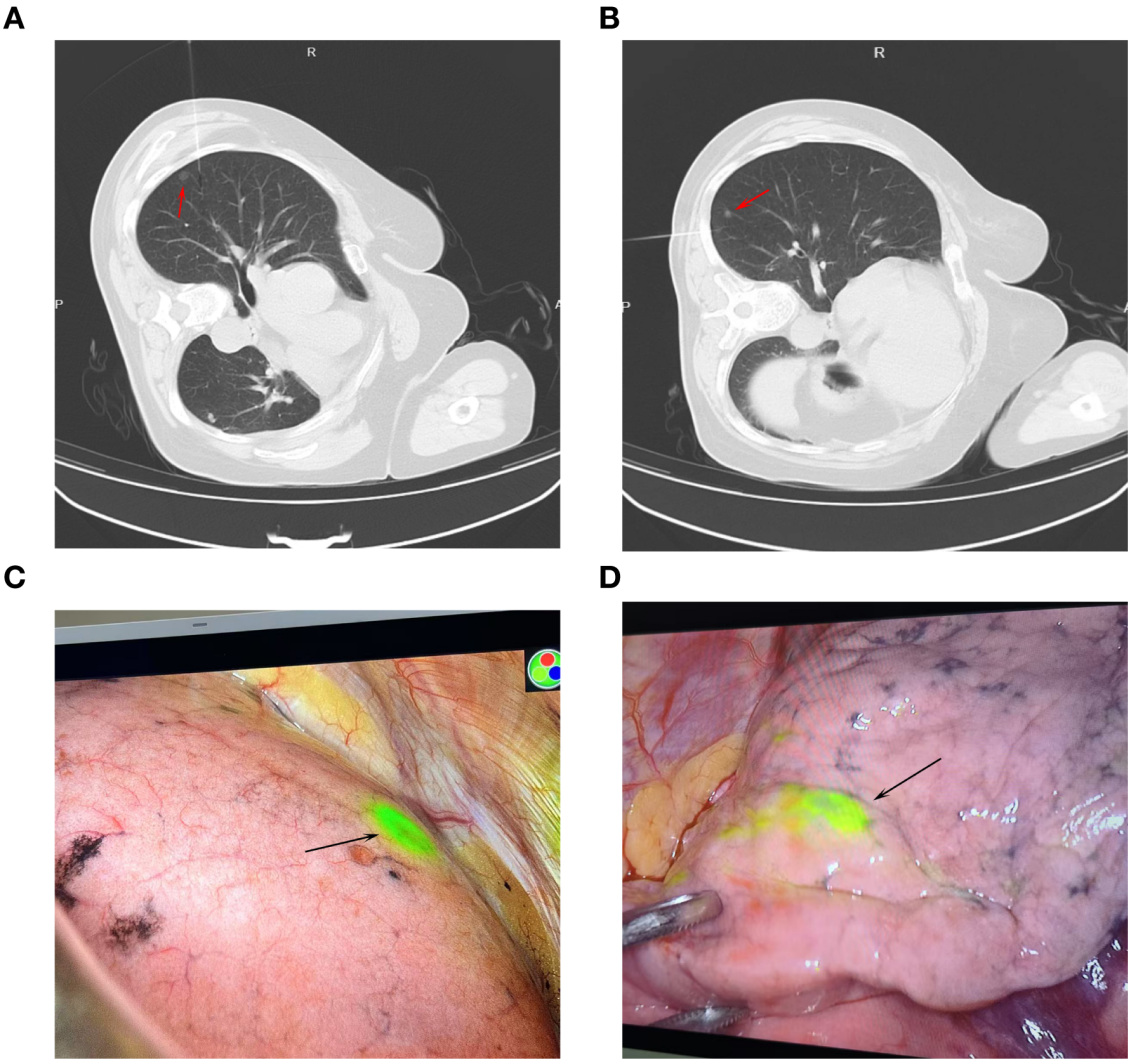
The CT-guided IG localization for PNs (arrows) in the right **(A)** upper and **(B)** lower lobes. **(C, D)** The IG localization (arrows) was found in the lung surface during the VATS.

Thirty-six cases with 79 PNs were enrolled in the SHA group (**Figure 1**) and 38 with 92 PNs represented the IG group (**Figure 2**). All PNs were localized preoperatively under CT guidance before VATS. Baseline demographic and clinical data were comparable between the two cohorts (**Table 1**).

**Table 1:** Baseline characteristics between 2 groups.

Variables	Soft hook anchor group	Indocyanine green group	P
Patients number	36	38	
Age (y)	56.1 ± 9.0	53.1 ± 12.4	0.239
BMI (kg/m2)	23.7 ± 2.8	22.7 ± 2.9	0.131
Gender			0.506
Male	11	9	
Female	25	29	
Number of PNs			0.216
2	30	25	
3	5	10	
4	1	3	
Diameter (mm)	7.7 ± 2.3	7.1 ± 3.1	0.101
PN-pleura distance (mm)	10 (Q1: 5; Q3: 17)	11.3 (Q1: 3.8; Q3: 21)	0.454
Nature of the PNs			0.736
Solid	18	19	
Ground glass	61	73	
Sides of the lung			0.219
Left	23	35	
Right	56	57	
Lobes			0.428
Upper	46	48	
Non-upper	33	44	

### Localization outcomes

CT-guided localization using both SHA and IG was technically successful in all patients, yielding a 100% success rate for each method. Each patient underwent one session of localization. However, the mean localization time was markedly less with IG relative to SHA (10.6 ± 4.4 minutes vs. 23.4 ± 11.9 minutes, P = 0.001), indicating a more efficient procedure with IG (**Table 2**). The time interval between localization and VATS was comparable between SHA and IG groups (146.9 ± 59.3 minutes vs. 148.6 ± 56.2 minutes, P = 0.899).

**Table 2:** Comparison of localization-related data.

Variables	Soft hook anchor group	Indocyanine green group	P
Successful localization rate	100%	100%	
Duration of localization (min)	23.4 ± 11.9	10.6 ± 4.4	0.001
Body position change	17 (47.2%)	12 (31.6%)	0.168
Time interval between localization and VATS (min)	146.9 ± 59.3	148.6 ± 56.2	0.899
Complications			
Pneumothorax	10 (27.8%)	14 (36.8%)	0.405
Lung hemorrhage	14 (38.9%)	8 (21.1%)	0.093

VATS: video−assisted thoracoscopic surgery.

Pneumothorax development was similar in the two groups (27.8% for SHA vs. 36.8% for IG, P = 0.405). All cases of pneumothorax were mild, required no additional intervention, and did not interfere with the subsequent surgical procedure. Univariate logistic regression analysis identified the presence of more than two nodules (P = 0.057), upper lobe location (P = 0.071), body position change during the procedure (P = 0.003), and prolonged localization time (P = 0.072) as factors potentially associated with pneumothorax. On multivariate analysis, only body position change remained a statistically significant independent risk factor (P = 0.03).

Lung hemorrhage occurred in 38.9% of cases in the SHA group and 21.1% in the IG group; however, this difference was non-significant (P = 0.093). All hemorrhages were limited in extent and did not impact VATS resection. Univariate analysis suggested that both longer localization time (P = 0.008) and use of SHA (P = 0.011) were linked to a greater likelihood hemorrhage. However, these factors were not found to be significant in the multivariate analysis (P = 0.099 and 0.262, respectively).

### VATS outcomes

Limited resection via VATS was technically successful in all cases across both groups (**Table 3**). All patients underwent one-stage resection of multiple PNs. In the SHA group, 54 nodules underwent wedge resection and 25 were managed with segmentectomy. In the IG group, 66 nodules were resected via wedge resection and 26 by segmentectomy. Additional lobectomy was performed in nine cases who underwent SHA and three who underwent IG due to intraoperative confirmation of invasive adenocarcinoma. Median operative time for VATS was comparable in the two groups (85 minutes vs. 80 minutes, P = 0.269). Final pathological diagnoses were also comparable, with no significant intergroup differences (P = 0.059).

**Table 3:** Comparison of VATS related data.

Variables	Soft hook anchor group	Indocyanine green group	P
Technical success of limited resection	100%	100%	
Limited resection types			0.630
Wedge resection	54	66	
Segmental resection	25	26	
Additional lobectomy	9	3	0.046
Duration of VATS (min)	85 (Q1: 60; Q3: 135)	80 (Q1: 62; Q3: 95)	0.269
Final diagnoses			0.059
Invasive adenocarcinoma	12	3	
Mini-invasive adenocarcinoma	25	33	
Adenocarcinoma in situ	14	17	
Metastasis	0	2	
Benign	28	37	

VATS: video−assisted thoracoscopic surgery.

## Discussion

Managing multiple pulmonary nodules surgically poses a significant challenge, as it requires the complete removal of all targeted lesions while preserving as much lung function as possible. To minimize loss of pulmonary capacity, multiple limited resections are preferred. Performing all resections in a single session may also reduce the risk of tumor progression compared to staged procedures.

Here, the effectiveness and safety profiles of two preoperative localization techniques under CT guidance, SHA and IG, were compared in patients undergoing VATS resection of multiple PNs. Both methods demonstrated excellent localization success, achieving a 100% technical success rate, consistent with previously reported rates for SHA (97.7%–98.9%) and IG (100%) (10–12). SHA’s effectiveness can be attributed to the anchoring capability of its four-hook design, which ensures stability within the lung parenchyma, and the tri-colored suture, which facilitates intraoperative visualization. In contrast, the key to IG localization lies in accurate dosing. As Lin et al. (9) reported, injecting 0.3 ml of IG strikes an optimal balance: larger volumes may lead to leakage and diffusion, while smaller amounts can result in inadequate marking.

The localization procedure was significantly faster with IG, which aligns with Lin et al.’s findings comparing IG with hook-wire techniques (11.2 ± 5.5 min vs. 14.9 ± 6.4 min, P = 0.003) (9). The relative simplicity of IG, as a liquid-based marker, likely contributes to its shorter procedural time compared to solid devices like coils, wires, and SHA.

While both techniques had similar rates of pneumothorax and lung hemorrhage, these complications were generally mild and did not delay or disrupt subsequent surgical intervention. Multivariate analysis identified intra-procedural changes in patient positioning as an independent risk factor for pneumothorax, corroborating previous studies focused on multiple-nodule localization (10, 13). No independent predictors of hemorrhage were identified, possibly due to the limited sample size.

Many researchers considered that the safe time interval between localization and VATS was less than 3 hours (6, 8, 9). In this study, the time interval between localization and VATS were comparable between 2 groups, and the mean time interval in both groups (146.9 ± 59.3 and 148.6 ± 56.2 minutes) were less than 3 hours. Prolonged time interval between localization and VATS may increase the risk of the delayed localization-related complications, which may influence the VATS procedures.

VATS outcomes were nearly identical in terms of operative time, resection type, and pathological findings. Although both localization methods facilitated accurate resection, each has unique advantages and limitations. IG, being deposited on the lung surface, relies on preoperative CT to estimate lesion depth. In contrast, SHA offers tactile feedback through the palpation of its anchor and visual cues from its colored suture, allowing more precise intraoperative depth assessment (10).

Other localization techniques, such as coils and radiotracers, have also been explored. Coil placement can be technically demanding due to its configuration and the requirement that one end be anchored near the lesion while the other protrudes beyond the pleura (11). Although radiotracer-based methods are less technically complex, they require intraoperative fluoroscopy, which increases radiation exposure for both patients and surgical staff (14).

This study has several limitations. First, its retrospective nature may have led to selection bias. Second, procedural variations between centers and operator experience could have affected outcomes. Finally, the sample size was relatively small, as cases involving multiple PNs represent only a subset of the broader population undergoing localization. Larger, prospective, multi-center randomized studies are required for verification of the results.

## Conclusion

In summary, both SHA and IG localization under CT guidance are effective and safe for the preoperative evaluation of multiple PNs. While both methods demonstrated excellent localization and surgical outcomes, IG localization was associated with significantly shorter procedural time. Nonetheless, each approach has specific advantages that may guide clinical decision-making. These results should be verified through larger, prospective, multi-institutional studies.

## Data Availability

The raw data supporting the conclusions of this article will be made available by the authors, without undue reservation.
